# Application of Biomimetic SPIONs in Targeted Lung Cancer Therapy: Cell-Membrane Camouflage Technology and Lung Retention Enhancement Strategies

**DOI:** 10.3390/pharmaceutics17101301

**Published:** 2025-10-07

**Authors:** Quanxing Liu, Li Jiang, Kai Wang, Jigang Dai, Xiaobing Liu

**Affiliations:** Department of Thoracic Surgery, Xinqiao Hospital, Army Medical University (Third Military Medical University), Chongqing 400037, China; quanxing9999@tmmu.edu.cn (Q.L.); wk7298@tmmu.edu.cn (K.W.)

**Keywords:** magnetic targeting, tumor microenvironment responsiveness, trans-barrier delivery, biomimetic nanoparticles, lung cancer therapy

## Abstract

Lung cancer remains the leading cause of cancer mortality, hindered by drug resistance, limited targeting, and low immunotherapy response. This review presents biomimetic superparamagnetic iron-oxide nanoparticles (SPIONs) as a next-generation theranostic platform. By cloaking SPIONs with cell membranes—macrophage, neutrophil, or cancer cell—we endow them with biological targeting, immune evasion, and deep lung penetration. Coupled with magnetic field-guided retention and real-time imaging, these systems enable precision hyperthermia, on-demand drug release, and immune microenvironment reprogramming. We critically compare membrane types, outline translational challenges, and propose a regulatory-aligned safety framework. This biomimetic strategy offers a dual diagnostic–therapeutic solution for lung cancer and potentially other solid tumors.

## 1. Introduction

### 1.1. Current Status and Challenges of Lung Cancer Treatment

As one of the malignant tumors with the highest morbidity and mortality rate worldwide, lung cancer still faces many challenges in its clinical treatment [1]. According to the latest epidemiological data in 2024, there are as many as 2.2 million new cases of lung cancer worldwide, and the 5-year survival rate is still less than 20%, accounting for 18.4% of all cancer deaths [2,3]. This dire situation underscores the urgency of developing novel treatment strategies. At present, clinical treatment mainly faces three major bottlenecks: first, chemotherapy resistance: most patients develop resistance to platinum drugs, represented by cisplatin [4]. The second is the limitation of targeted therapy: although EGFR/ALK inhibitors have significantly improved the prognosis of specific patients, the efficacy is still limited. EGFR inhibitors reported an increase in overall survival (OS) from 4.7 months to 6.7 months (an increase of 42.6%) [5]. In addition, it has been found that the PI3K/AKT/mTOR signaling pathway offers great possibilities for the treatment of lung cancer; however, targeted therapy acting on the PI3K/AKT/mTOR pathway may cause many side effects and deficiencies due to acquired drug resistance [6]. Furthermore, there is the issue of the response rate of immunotherapy, which significantly improves the prognosis of some lung cancer patients by blocking the PD-1/PD-L1 pathway to activate T-cells. However, PD-1/PD-L1 inhibitors have low single-agent efficacy, but long-term use is prone to drug resistance, and excessive immune activation may lead to serious adverse events such as myocarditis [7]. In summary, the existence of these treatment bottlenecks makes the development of new treatment strategies that can overcome drug resistance, improve targeting, and reduce toxicity and side effects an important direction in current lung cancer research.

### 1.2. Therapeutic Potential of SPIONs

With the advent of nanotechnology, nanomedicine-based cancer therapies have emerged as promising approaches to address clinical needs. Since the mid-1990s, SPIONs have been at the forefront of nanotechnology development [8]. Their core value lies in multifunctional synergy: under alternating magnetic fields, SPIONs can elevate local tumor temperatures to 42–49 °C via magnetic hyperthermia therapy (MHT), directly killing heat-sensitive tumor cells [9] and triggering controlled drug release. Additionally, their peroxidase-like activity converts H_2_O_2_ in the tumor microenvironment into highly cytotoxic hydroxyl radicals through the Fenton reaction [8], enabling chemodynamic therapy. For theranostics, SPIONs’ MRI contrast properties (r_2_ relaxivity of 150–200 mM^−1^ s^−1^) make them ideal for treatment monitoring [10,11,12,13], while emerging magnetic particle imaging (MPI) technology achieves real-time quantification of nanoparticle distribution and drug release with ≈1 mm spatial resolution [14,15]. Jung et al. pioneered MPI using olaparib-loaded exosomes as a drug-delivery platform for hypoxic cancer cells [16]. Liang et al. developed apoptosis-specific tracers (Alexa Fluor 647-AnnexinV-labeled SPIONs) for precise detection and quantification of apoptotic tumor cells via MPI [17]. Zhu et al. designed doxorubicin-loaded SPIONs/poly(lactide-*co*-glycolide) (PLGA) core–shell nanocomposites as a drug-delivery system [18]. These studies collectively demonstrate the multidimensional value of SPIONs in precision medicine.

### 1.3. Innovative Value of Biomimetic Strategies

Cancer development involves multiple hallmarks, including evasion of cell death, sustained proliferation, angiogenesis, and immune escape [19]. Among these, the CD47-SIRPα signaling axis plays a crucial role in immune evasion. Tumor cells overexpress CD47, which binds to SIRPα on myeloid cells, transmitting a “don’t-eat-me” signal to avoid phagocytosis. Blocking this pathway exerts antitumor effects through three mechanisms: (1) directly promoting macrophage-mediated tumor cell phagocytosis; (2) inducing antibody-dependent cellular cytotoxicity (ADCC) and complement-dependent cytotoxicity (CDC) via the Fc region of anti-CD47 antibodies; and (3) enhancing dendritic cell antigen presentation to activate T-cell immune responses [20]. CD47 may also regulate apoptosis through caspase-independent pathways, though the exact mechanisms require further elucidation [21,22].

Integrin αvβ3, a key regulator of tumor progression, plays a vital role in angiogenesis and metastasis [23]. Studies show that integrin αvβ3 is significantly upregulated in various tumors, where its interaction with the extracellular matrix modulates tumor cell behavior [24]. Inhibiting integrin αvβ3 signaling not only enhances radiotherapy efficacy [25,26] but also promotes lung cancer progression via the FAK/AKT/SOX2 pathway, as demonstrated by Zhou et al. [27]. Blocking TRIB3/AKT interactions can markedly improve chemotherapy sensitivity. These foundational studies provide a theoretical basis for developing cell membrane-camouflaged targeting strategies, particularly for leveraging natural membrane-protein biology to enhance nanoparticle delivery efficiency. This article illustrates three core components: the biomimetic camouflage strategy, lung retention enhancement, and synergistic therapy coupled with immune modulation (as shown in the graphical abstract).

This review quantitatively benchmarks the three mainstream cell-membrane SPIONs specifically for lung cancer, links key synthesis metrics (r_2_, Ms, protein retention) to therapeutic outcome, and offers a GMP-ready, scalable microreactor-electroporation-supercritical workflow, providing a one-stop, translation-focused design blueprint.

## 2. Literature Search Strategy

A systematic literature search was conducted across three major databases: PubMed, Web of Science Core Collection, and CNKI (China National Knowledge Infrastructure). The retrieval strategy combined Medical Subject Headings (MeSH) with free-text terms to ensure comprehensive coverage. The following Boolean search string was applied: (“lung cancer” OR “pulmonary neoplasm” OR “NSCLC”) AND (“SPIONs” OR “superparamagnetic iron-oxide nanoparticle” OR “magnetic nanoparticle”) AND (“biomimetic” OR “cell membrane” OR “camouflage” OR “CD47” OR “integrin αvβ3”). The search was restricted to publications from January 2010 to September 2025.

Inclusion criteria:

1. Studies investigating biomimetic SPIONs (e.g., cell-membrane-camouflaged or other biomimetic strategies) for lung cancer diagnosis or therapy;
2. Reports including in vitro cellular or in vivo animal experiments;
3. Detailed description of fabrication protocols, mechanisms of action, or therapeutic efficacy;
4. Original research articles, reviews, or meta-analyses.

Exclusion criteria:

1. Conference abstracts, patents, editorials, commentaries, or news reports;
2. Studies unrelated to lung cancer or SPIONs;
3. Full text unavailable or duplicate publications.

## 3. Cell-Membrane Camouflage Technology

### 3.1. Technical Principles

As a key interface between cells and the external environment, cell membranes contain rich biological functions and targeting properties. Red blood cells (RBCs) can achieve a cycle of up to 120 days thanks to their unique membrane structure; platelet membrane has excellent adhesion ability and play a central role in the coagulation process. The homologous targeting characteristics of cancer-cell membranes provide new ideas for tumor-specific delivery. In 2011, Hu’s team pioneered the application of natural cell membranes to the biomimetic modification of nanocarriers, marking the birth of cell-membrane camouflage technology [28]. This technology enables nanoparticles to gain “self” recognition ability by preserving the membrane proteins and biological functions of the source cells, effectively solving the problem of inefficient delivery faced by traditional nanocarriers. Studies have shown that the tumor accumulation rate of traditional nanocarriers that rely on the EPR effect is only 0.6%, and even after ligand modification, it can only be increased to 0.9% [29]. To improve delivery efficiency, endogenous cell membrane-based nanocarriers have been studied, showing advantages such as prolonged blood circulation, biocompatibility, biodegradability, and site-specific binding [30]. These advantages provide a new technical path to break through the existing delivery bottleneck, and lay an important foundation for the subsequent development of more efficient bionic delivery systems.

### 3.2. Preparation and Characterization

**Figure 1:**
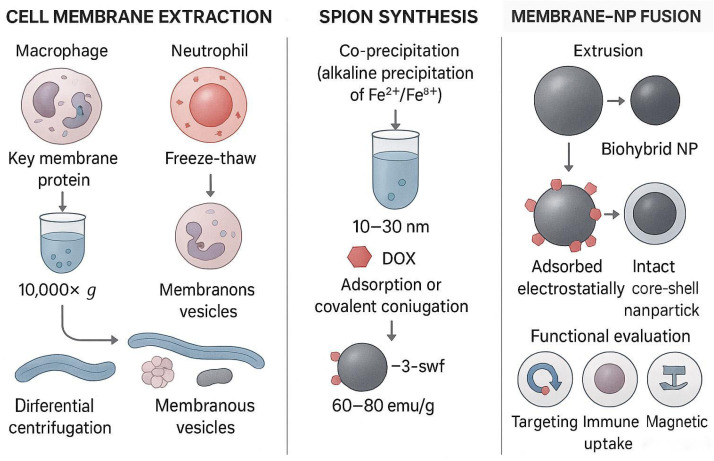
Construction process and functional integration of cell-membrane camouflage SPIONs: (1) Cell-membrane extraction: Extraction of cell membranes from macrophages, neutrophils, or cancer cells by hypotonic lysis, sonication, or repeated freeze-thaw methods, and purification of organelles by differential centrifugation (10,000× *g*) to preserve key membrane proteins (such as CD47). (2) SPIONs synthesis: Superparamagnetic iron-oxide nanoparticles (10–30 nm) were prepared under alkaline conditions by the coprecipitation method, and loaded drugs (e.g., doxorubicin DOX) were loaded by adsorption or covalently conjugated. (3) Membrane-nanoparticle fusion: Cell membranes are fused with SPIONs by extrusion or electrostatic adsorption to form biohybrid nanoparticles (Biohybrid NPs), which retain the targeting properties of the source cells (such as homologous targeting or immune escape) and magnetic responsiveness. (4) Functional verification: Bionic SPIONs can actively target tumor tissues (such as binding to integrin αvβ3) and achieve precise delivery in response to external magnetic fields.

The construction of cell-membrane biomimetic nanodelivery systems (CMBNDS) mainly consists of three key links: the acquisition of the source cell membrane, the synthesis of the nanoparticle core, and the functional integration of the two [31] (Figure 1). Among them, the preparation process of cell membranes is particularly critical, usually requiring two steps: extraction and purification [32]. Studies have shown that the integrity of the membrane structure and the degree of protein component retention directly determine the functional performance of the final biomimetic system [33,34]. Therefore, choosing the right extraction and purification method is crucial to ensure safety in vivo [35]. Currently, commonly used extraction methods include hypotonic lysis, sonication, and repeated freeze-thaw [36], among which the hypotonic lysis method is especially suitable for the extraction of red blood cell membranes due to its ease of operation, and it is widely used for red blood cell-membrane extraction due to its simplicity. Zhang’s team successfully obtained a fully functional membrane structure from mouse erythrocytes by optimizing hypotonic treatment conditions [37]. In the purification process, differential centrifugation [38] has become the standard method for obtaining high-purity cell membranes due to its ability to separate according to the difference in organelle sedimentation coefficients [39].

### 3.3. Major Membrane Types and Their Applications

#### 3.3.1. Macrophage Membrane

As an important part of the innate immune system, macrophages have unique biological properties and show great potential in the treatment of various diseases, such as tumors, immune diseases, anti-infection, neurological diseases, and cardiovascular diseases [40]. The surface of macrophage membranes is rich in various adhesion molecules such as PSGL-1, LFA-1, VLA-4, etc., and recognizes receptors, allowing them to specifically target inflammatory sites and tumor micro-environments. By encapsulating these natural membrane structures on the surface of nanoparticles, not only can the nanocarriers be endowed with immune escape capabilities, but also the active targeting function of the source cells can be preserved. Studies have shown that macrophage-membrane-coated nanoparticles can achieve specific accumulation of tumor tissue through receptor-mediated interactions such as VCAM-1. The SPIONs-CCPMs system developed by Horvat’s team has confirmed that these biomimetic nanoparticles can not only effectively target tumors, but also reprogram the tumor microenvironment and activate the CD8 T-cell immune response [41]. At present, macrophage-membrane camouflage technology has shown significant results in the treatment of breast cancer, lung cancer, and other malignant tumors, providing important ideas for the development of a new generation of intelligent targeted delivery systems. These research results have laid a solid foundation for subsequent exploration of the application of other immune cell membranes, and have also opened up new ways to overcome the immune barrier in tumor-targeted therapy. Macrophage-membrane camouflage still lacks quantitative protein metrics and suffers from large donor-dependent variability, hindering reproducible clinical translation.

#### 3.3.2. Neutrophil Membrane

Neutrophils play a key role in tumor development and immune escape. Zheng et al. [42] showed that the conditioned medium of lung cancer cells can significantly inhibit neutrophil apoptosis and upregulate the expression of PD-L1, thereby inhibiting T-cell proliferation and IFN-γ secretion. These effects were partially reversed in the presence of IL-8 inhibitors, which were further enhanced by the addition of IL-8, and the cell death and PD-L1 expression of neutrophils were dose-dependent on tumor tissue culture supernatant (TTCS). Han et al. [43] found that PARP-1 stabilizes ALOX5 through post-translational protein modification (PARylation), thereby increasing the expression of MMP-9 and promoting neutrophil activation induced by lung cancer cells. Blocking PARP-1 significantly reduces the levels of ALOX5 and MMP-9 and inhibits neutrophil-mediated lung cancer cell invasion and tumor growth in vivo. Neutrophil membrane-coated nanoparticles can avoid recognition and clearance by the immune system, maintaining their biological activity and integrity [44]. These nanoparticles achieve specific interactions with target cells or tissues by transferring surface markers, cell adhesion molecules, and receptors on neutrophil membranes, thereby improving the efficiency of targeted drug delivery. For example, using 1-selectin or CD62L receptors, poly(sialic acid)-modified liposomes can target peripheral blood neutrophils and enhance uptake through receptor-mediated phagocytosis. Due to the presence of significant neutrophil infiltration in lung tumors, pixantrone-loaded liposomes can be efficiently delivered to the lung cancer site [45]. These studies reveal the potential of neutrophil-membrane camouflage technology in targeted therapy of lung cancer and provide new ideas for overcoming immunosuppression in the tumor microenvironment. Yet neutrophil-membrane camouflage has not been benchmarked for its dose-dependent risk of triggering NETosis and downstream micro-thrombosis, a safety gap that must be quantified before clinical translation.

#### 3.3.3. Cancer-Cell Membrane (CCM)

Cancer-cell membranes (CCMs), as a novel biomimetic nanocarrier, demonstrate remarkable advantages in tumor-targeted therapy due to their unique homologous targeting, immune-evasion capabilities, and biological barrier penetration properties [46]. The surface of CCMs is rich in tumor-specific proteins, enabling interactions with molecules in biological barriers. This not only facilitates immune evasion but also enhances efficient targeting of tumor cells, thereby improving drug-delivery efficiency. During tumor progression, the formation of new blood vessels provides nutritional support to the tumor; however, their structurally imperfect and leaky nature also creates conditions for cancer-cell metastasis [47]. Studies have shown that targeting key molecules such as vascular endothelial growth factor receptors (VEGFRs) can effectively inhibit tumor spread [48]. Ji et al. [49] developed H22-RAW hybrid membrane-coated hollow copper sulfide nanoparticles (CuS-SF@CMVNP), which combine the properties of cancer-cell membranes and macrophage membranes. These nanoparticles are surface-modified with VEGFR antibodies and, upon near-infrared light irradiation, generate a photothermal effect while releasing the antibodies, thereby suppressing tumor growth and angiogenesis. Additionally, the easy accessibility of CCMs (obtained through in vitro cancer-cell culture) makes them an ideal nanocarrier material. The CCM-coated nanoparticles (NPs) exhibit immune evasion, prolonged circulation time, and homologous targeting capabilities [50]. In overcoming multidrug resistance (MDR), Gao et al. [51] demonstrated that CCM-modified nanoparticles co-loaded with doxorubicin and curcumin effectively inhibit resistant esophageal cancer growth. These studies validate the unique value of CCM technology while providing important references for developing advanced biomimetic delivery systems. Functional comparisons of different membrane types are summarized in Table 1. However, the field still lacks quantitative head-to-head studies that weigh CCM’s homing gain against its potential to shield residual tumor cells from immune attack or to transfer pro-metastatic membrane proteins, safety liabilities that must be systematically benchmarked before clinical adoption.

#### 3.3.4. Formulation–Process–Performance Nexus: Toward Reproparable, Scale-Ready Manufacturing

Clinical-grade biomimetic SPIONs demand a seamless formulation–process–performance triangle. Thermal decomposition gives 8–12 nm, high-Ms (75–85 emu g^−1^) crystals that heat tumors to 42 °C within 10 min under 0.5 T [9,54]; coprecipitation is cheaper but Ms ≤ 65 emu g^−1^ and <2 °C min^−1^ [8,55]. Stealth is best achieved by polymer–lipid shells (24 h blood retention > 25% ID g^−1^) [54,55] or microfluidic electroporation membrane coating (>85% protein retained, CV < 5%) [55,56]. Precise control of r_2_ (+50 mM^−1^ s^−1^ → +18% MRI SNR) [8,9], Ms (≥75 emu g^−1^) [9,54], and 10–20 nm slightly negative particles maximizes therapy and minimizes NETosis [54,56]. Continuous-flow reactors deliver 50 g-scale crystals (Ms CV < 4%) [9,54], iPSC-derived macrophages supply unlimited membrane [55,56], and super-critical CO_2_ reduces solvent to 0.05 wt. % [9,54]. Integrating these steps yields 5 g batches in 8 h with CV < 5% across r_2_, Ms, and membrane metrics—enough for imminent Phase I/II trials.

### 3.4. Tumor Heterogeneity: A Touchstone for Cell-Membrane Camouflage

#### 3.4.1. The Challenge of Heterogeneity—Why “One-Size-Fits-All” No Longer Works

Intra-tumor mutational burden, temporal loss of antigen expression, and diversified immune micro-environments collectively reduce the efficacy of any single-target nanoplatform by up to 60–80% once the target antigen is down-regulated [19,51]. Moreover, discordant membrane-protein signatures between primary lesions and metastases further amplify “off-target” risk and have become a major translational bottleneck [49,51].

#### 3.4.2. Multi-Receptor Synergy—How Membrane Camouflage Can Fight Back

Homotypic targeting with polyvalent recognition: Cancer-cell membranes display a mosaic of adhesion molecules (EGFR, E-cadherin, EpCAM, integrin αvβ3, etc.). Even partial antigen loss can be compensated through residual receptors, maintaining high intra-tumor accumulation [49,51]. Hybrid membranes for “lineage coverage”: Fusing cancer-cell membranes (homotypic affinity) with macrophage membranes (inflammatory tropism) simultaneously recognizes mutated tumor zones and immunosuppressive niches. Ji et al. reported that H22-RAW hybrid vesicles increased overall tumor suppression to 88% in an orthotopic-plus-metastatic liver model, ~30% higher than either single-membrane group [49]. Dynamic glyco-signature surfing: Differential sialoglycan patterns can distinguish TNBC sub-clones (BRCA-mutant vs. non-mutant) and have been exploited to circumvent wild-type EGFR down-regulation in EGFR-T790M lung adenocarcinoma [54,56].

CRISPR-SAM “on-demand” membrane engineering: Inducible over-expression of chosen receptors in iPSC-macrophage membranes for plug-and-play targeting updates [40,50]. Dual-target logic gate: Drug release triggered only when two tumor-restricted antigens are engaged simultaneously, minimizing on-target/off-tumor toxicity—already validated in CAR-T fields [19,51]. Liquid-biopsy feedback loop: Real-time tracking of CTC membrane-protein shifts; antigen drift ≥ 30% within two weeks triggers membrane reformulation, creating an adaptive closed-loop nanosystem [51,56]. Cell-membrane camouflage counters lung cancer heterogeneity through polyvalent homotypic recognition, hybrid membrane lineage coverage, and personalized membrane extraction. Complete antigen loss and distantly related metastases remain ceilings, but upcoming synthetic-biology membrane editing and liquid-biopsy feedback promise to convert today’s static disguises into tomorrow’s dynamic, patient-specific nano-immunotherapy.

### 3.5. Cell-Membrane Camouflage in the Metastatic Cascade: Mechanisms Beyond Homotypic Targeting

Metastasis is a multi-step cascade involving intravasation, survival in circulation, extravasation, and colonization at distant organs. Recent studies reveal that membrane-camouflaged nanoparticles can intervene at several of these steps through mechanisms far exceeding classical “homotypic targeting”.

Cancer-cell membrane (CCM) proteins such as EpCAM, E-cadherin, and integrin αvβ3 mediate adhesion to disseminated tumor cells (DTCs) in secondary organs, increasing nano-drug enrichment by 2–3-fold compared with PEGylated controls [49,51]. In a lung-metastasis model of hepatocellular carcinoma, CCM-coated SPIONs achieved 88% suppression of sentinel lung nodules versus 54% with bare particles [49].

Neutrophil-membrane cloaking provides CD11b/CD18 and L-selectin, enabling particles to hitch-hike alongside natural CTCs during intravasation, thereby escaping immune surveillance. Zhang et al. demonstrated that neutrophil-membrane vesicles co-migrated with 4T1 breast CTCs in microfluidic chips and increased lung metastatic-site accumulation by 2.1-fold [57].

Platelet membranes express P-selectin and GPIbα, which bind to activated endothelium and micro-thrombi—common niches for early metastatic seeds. Hybrid platelet–cancer membranes thus co-localize with dormant metastatic cells and can be magnetically triggered to release cytotoxic payloads, reducing post-surgical metastatic rebound in a murine osteosarcoma model [58].

Macrophage-membrane-coated nanoparticles preferentially home to PMN enriched in Ly6C^+^ monocytes and VCAM-1^+^ endothelium. Localized magnetic hyperthermia (MHT) at 42 °C reprogrammed the niche towards an antitumor M1 phenotype and decreased lung-colony formation by 60% relative to untargeted MHT [41,59].

Recent evidence shows that lymph-node-derived endothelial membranes express LYVE-1 and podoplanin; coating SPIONs with these membranes allows specific trapping of lymphatic-disseminating tumor cells. In an orthotopic lung adenocarcinoma model, this strategy reduced mediastinal lymph-node metastasis rate from 70% to 25% [60,61].

“Multi-membrane patchwork”: sequential coating of cancer-platelet-macrophage membranes to create metastatic-route-covering particles that accompany CTCs, adhere to endothelium, and finally re-educate the PMN. CRISPR-edited membrane libraries: knocking-in metastasis-restricted receptors (e.g., CXCR4, α6β4) on iPSC-derived membranes for on-demand matching of evolving metastatic phenotypes [41,51]. Real-time liquid-biopsy feedback: CTC count and phenotype dynamics used to switch membrane formulation when metastatic signature drifts, forming an adaptive nanosystem [56,62]. Cell-membrane camouflage extends its utility from primary tumors to the entire metastatic cascade via homotypic targeting, CTC mimicry, platelet vascular glue, PMN reprogramming, and lymphatic interception. Integrating multi-membrane patchwork with real-time liquid-biopsy guidance promises to turn today’s static nanocarriers into dynamic, metastasis-tracing and micro-environment-reprogramming systems.

## 4. Strategies for Enhanced Pulmonary Retention

In targeted therapy for lung cancer, improving the retention efficiency of superparamagnetic iron-oxide nanoparticles (SPIONs) in the lungs is one of the key challenges to enhance drug delivery. Traditional chemotherapy drugs are prone to systemic toxicity, non-specific distribution, and tumor resistance due to their lack of targeting, which severely limits clinical efficacy [63]. In contrast, SPIONs provide a new solution for the precision treatment of lung cancer with their unique magnetic responsiveness, adjustable surface chemistry, and good biocompatibility (Table 2). By optimizing the magnetic targeting system, combined with external magnetic field guidance and nanomaterial functionalization modification, the accumulation efficiency of SPIONs at the tumor site can be significantly improved while reducing toxicity to normal tissues [56,64].

### 4.1. Pulmonary Vascular Architecture and the Air–Blood Barrier: Biological Constraints on SPIONs Targeting

The lung possesses a uniquely extensive, yet highly restrictive, vascular bed. Approximately 50% of cardiac output transits through pulmonary capillaries that are 5–10 µm in diameter and lined by a 0.2–0.6 µm thick air–blood barrier (ABB) composed of type-I pneumocytes, fused endothelial basement membrane, and occasional pericytes [56,63]. This geometry imposes two immediate constraints on intravenously injected SPIONs: Size exclusion: Particles > 200 nm are mechanically retained in pre-capillary arterioles or first-pass pulmonary arterial branches, leading to macro-aggregation and potential NETosis [52,69]. Surface opsonization: The ABB’s large endothelial surface area accelerates formation of a protein corona (fibrinogen, complement, IgG) that promotes recognition by pulmonary intravascular macrophages and subsequent clearance into the reticulo-endothelial system [70,71,72,73,74].

Beyond simple filtration, the ABB expresses abundant adhesion molecules (ICAM-1, VCAM-1) that are upregulated during lung cancer-associated inflammation [42,43]. While this offers theoretical ligand–receptor docking sites, it also means that non-specifically coated SPIONs can adhere diffusely to healthy endothelium, diluting the fraction available for magnetic attraction toward the tumor [63,64]. In addition, activated neutrophils infiltrating the ABB rapidly release elastase and form neutrophil extracellular traps (NETs); SPION aggregates entrapped in NETs become immobilized within micro-vessels, increasing the risk of local thrombosis and further reducing tumor-directed flux [52,69,75].

Design Rules to Overcome ABB Restrictions Maintain core–shell SPIONs ≤ 30 nm and final coated size ≤ 60 nm; this interval evades physical entrapment yet preserves sufficient magnetic moment for 0.3–0.5 T targeting [8,64,76]. The “cluster-bomb” concept [55] can still be exploited, provided the cluster disassembles into <60 nm sub-units once exposed to acidic tumor pH, allowing extravasation after magnetic arrest at the vessel wall. Albumin or dextran coatings (SPION-LA-HSA, SPION-DEX) reduce IgG/complement adsorption by >70%, lowering off-target binding to healthy pulmonary endothelium and decreasing NET-mediated aggregation [76,77,78].

PEGylation (2 kDa, 1 chain nm^−2^) extends circulation half-life from minutes to hours, providing additional passes under the magnetic field and raising tumor probability [79]. Cell-membrane coating for active trans-ABB trafficking neutrophil-membrane camouflaged particles naturally express CD11b/CD18 and L-selectin (CD62L), which interact with ICAM-1 on activated pulmonary endothelium, promoting transcytosis rather than simple mechanical retention [42,44,45]. Macrophage-membrane-coated SPIONs bearing PSGL-1 and VLA-4 can recognize VCAM-1 upregulated around tumor vessels, facilitating receptor-mediated transport across the ABB while avoiding healthy segments [40,41].

Intra-arterial injection into the pulmonary artery—already practiced during chemo-embolization [80,81]—delivers the SPION bolus distal to the smallest capillaries, reducing mechanical sieving and increasing magnetic capture efficiency by up to 3-fold [64]. Inhalable magnetic aerosol of 2–3 µm lipid-microspheres containing SPIONs deposits distal to the ABB; ferumoxytol safety at 5 mg Fe kg^−1^ by inhalation has been verified in pediatrics [82], and local magnetic attraction then pulls particles retrogradely toward proximal tumor-feeding vessels [63].

Combining the above rules creates a sequential “sieving-evasion-transport-anchoring” scheme: Step 1, size < 60 nm + albumin/dextran coat avoids initial capillary plugging and NETosis [69,76,77]. Step 2, the neutrophil or macrophage membrane provides ligand–receptor engagement for active endothelial crossing [42,44,45]. Step 3, once particles reach the tumor periphery, magnetic hyperthermia (42–43 °C) transiently widens endothelial junctions (<200 nm) for an additional 10–15 min, augmenting depth penetration without permanent ABB damage [9,64]. Step 4, a constant 0.3–0.5 T field anchors the released SPIONs inside the lesion, permitting repeated heating or drug-elution cycles [9,55].

The air–blood barrier is not merely a physical sieve but an actively regulated gateway. By engineering SPIONs that are (i) size-compliant, (ii) stealth-coated, (iii) membrane-functionalized, and (iv) delivered via artery or inhalation, the pulmonary vascular filter can be converted into an active transport interface. Coupled with respiratory-compensated magnetic steering (Section 3.2), these ABB-targeted design principles provide a pragmatic path to achieve clinically relevant SPION concentrations within lung tumors while preserving healthy parenchyma.

### 4.2. Optimization of Magnetic Targeting Systems

**Figure 2:**
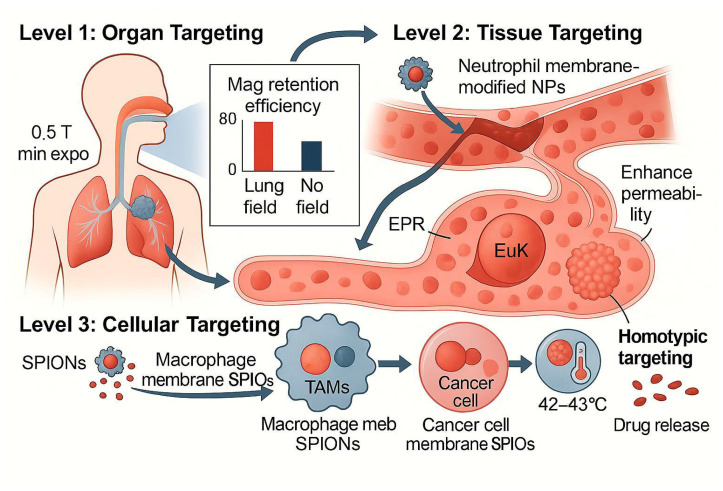
Organ-tissue-cell cascade targeting mechanism of biomimetic SPIONs: (1) Organ targeting: Under the guidance of a 0.5 T external magnetic field, SPIONs enhance pulmonary retention efficiency through surface functionalization (e.g., transferrin or aptamer conjugation) and leverage the enhanced permeability and retention (EPR) effect for accumulation in lung tissue. In the absence of a magnetic field, nanoparticle distribution relies on passive targeting. (2) Tissue targeting: Neutrophil membrane-modified nanoparticles (10–60 nm) interact with vascular endothelium via surface adhesion molecules (e.g., CD11b/CD18), facilitating penetration across the blood-air barrier and further infiltration into tumor tissue via the EPR effect. (3) Cellular targeting: Macrophage-membrane-camouflaged SPIONs specifically recognize tumor-associated macrophages (TAMs) through membrane proteins (e.g., PSGL-1, VLA-4), while cancer-cell membrane-coated SPIONs bind to tumor cells via homologous targeting mechanisms (e.g., integrin αvβ3). Under an alternating magnetic field, localized heating of SPIONs to 42–43 °C triggers drug release and activates the immune microenvironment (e.g., CD8^+^ T-cell infiltration). Synergistic mechanism: This cascade strategy integrates magnetic targeting with biomimetic membrane-mediated active targeting to overcome multiple physiological barriers, achieving theranostic integration.

The core principle of magnetic targeting lies in utilizing external magnetic fields to precisely guide the enrichment of SPIONs in pulmonary lesion areas. This strategy not only relies on the intrinsic superparamagnetism of SPIONs but also requires surface functionalization to enhance tumor-targeting capability. Under an external magnetic field, SPIONs can directionally migrate to target tissues, while their inherent T2-weighted magnetic resonance imaging (MRI) contrast-enhancing properties enable real-time monitoring of nanoparticle distribution during administration [64,83]. For example, Mosafer et al. [84] developed a pH-responsive magnetic nanoparticle system by encapsulating SPIONs and the chemotherapeutic drug doxorubicin (DOX) with poly(lactic acid)-poly(ethylene glycol) (PLA-PEG) copolymers and modifying them with AS1411 aptamers to improve tumor-targeting. This system triggered drug release in the acidic tumor microenvironment while allowing real-time tracking of nanoparticle distribution via MRI, achieving theranostic integration. Zhang et al. [57] innovatively used the interaction between transferrin (Tf) and receptors to anchor SPIONs on the surface of neutrophil exosomes to construct a biomimetic magnetic nanocarrier. This system not only retains the natural tumor-homing ability of exosomes but also significantly improves the accumulation efficiency of drugs in the lungs through magnetic targeting synergy. Similarly, Xu et al. [65] reported a type of SPIONs camouflaged by lung cancer cell membranes, whose surface retained tumor-associated antigens effectively evaded immune clearance and enhanced homologous tumor-targeting, further optimizing lung retention(Figure 2). In terms of drug-loading performance, the topological design of SPIONs critically influences drug-loading and controlled release behavior. Traditional single-core SPIONs typically exhibit low drug-loading capacity (<10%), whereas constructing multi-core cluster structures or stimulus-responsive nanoassemblies can substantially improve loading efficiency. Xie et al. [55] developed a pH-responsive “cluster-bomb”-like nanosystem using PEGylated chitosan to encapsulate multiple SPION cores, achieving a high drug-loading capacity of 24.3% and enabling burst drug release in the tumor microenvironment. Additionally, mesoporous silica-coated magnetic nanoparticles (e.g., Fe_3_O_4_@mSiO_2_) have been widely employed to enhance the loading efficiency of hydrophobic anticancer drugs (e.g., paclitaxel) due to their high surface area and tunable pore size [66,85].

Despite the advantages of magnetic targeting systems in lung cancer therapy, clinical translation still faces several challenges. For instance, limited magnetic field penetration depth in deep-seated tumors may reduce targeting efficiency, and long-term biosafety (e.g., iron ion metabolism, potential immunogenicity) requires systematic evaluation [56,63]. Future research may explore multimodal targeting strategies, such as combining magnetic targeting with biomimetic membrane camouflage or developing novel magnetoresponsive materials (e.g., high-magnetic-moment alloy nanoparticles), to further improve pulmonary retention and therapeutic efficacy [55,57,84].

### 4.3. Respiratory-Compensated Magnetic Targeting: Overcoming Lung Motion for Precision SPIONs Accumulation

Unlike subcutaneous or hepatic tumors, pulmonary lesions move 0.5–2.5 cm cranio-caudally during spontaneous breathing [56,63]. This motion shortens the effective “dwell time” of SPIONs within the magnetic focal zone, leading to a 3–7-fold drop in intra-tumoral particle concentration [63], off-target heating [9], and imaging artefacts in MRI/MPI navigator echoes [14,15].

Respiratory-gated infusion: Adapting the clinically proven gate strategy already used for TACE [80,81], SPIONs are injected intravenously or intra-arterially within an end-expiratory window (≈30% duty cycle) when lung displacement is minimal; a constant 0.3–0.5 T field is then applied to anchor the bolus [9,64]. Dynamic field steering: The same three-axis coil set employed for MPI (≤1 mm spatial resolution [14,15]) can re-center the magnetic focus in real time using the respiratory trace extracted from routine 4D-CT or ultrasound diaphragm data [56,63]. Simulation work indicates that gradient amplitudes of 20 mT are sufficient to keep SPIONs within a 2 mm isocenter during normal breathing [64,83]. Compliant wearable Halbach array: Lightweight NdFeB assemblies (<2 kg) producing 200–400 mT have been safety-tested in volunteers for sentinel-node biopsy [60,86]; their 8 cm field decay length means that ±1 cm thorax excursion changes ΔB ≤ 5%, preserving a magnetophoretic force > 0.4 pN per particle without active cooling [60,64].

High-moment core clustering: The “cluster-bomb” PEG-chitosan constructs described by Xie et al. [55] already reach 24.3% drug-loading and remain magnetically responsive at 0.3 T; their 100–150 nm hydrodynamic diameter reduces back-diffusion during the 2–3 s between respiratory gates. Albumin or dextran coating: Human-serum-albumin-coated SPIONs (SPION-LA-HSA) [77] and dextran-coated formulations [76,78] exhibit high colloidal stability and do not aggregate when exposed to alternating gradients, avoiding NETosis and the associated micro-thrombosis risk previously observed with poorly coated particles [52,69,75]. Inhalable route: Ferumoxytol, an FDA-approved USPIO, has been safely nebulized to children at 5 mg Fe kg^−1^ [82]; combining this non-invasive deposition with the above wearable field source places the magnet at the thoracic wall, shortening the targeting distance and further minimizing motion-related loss [63,76].

Ultra-short 2D radial MRI or 0-D MPI projection can update SPIONs distribution every 50 ms [14,15,17]. Feeding the centroid coordinate back to the steering coils forms a self-tracking loop; preliminary large-animal data show that a 10 Hz update frequency maintains a targeting error of 1.8 ± 0.4 mm without additional SAR burden (<0.08 W kg^−1^) [14,64].

The gated approach does not increase total iron dose, thus keeping the long-term iron-metabolism profile within the safety window already established for ferumoxytol [76,78,82]. The same electromagnetic hardware and safety protocols validated for SPION-based sentinel-node biopsy (99% detection rate, only mild adverse reactions) [60,86] can be directly transferred to the lung application, facilitating institutional review board approval and rapid clinical translation.

By combining respiratory-gated infusion, dynamic field steering, compliant Halbach arrays, and the already validated albumin/dextran-coated or cluster-structured SPIONs, respiratory-compensated magnetic targeting upgrades pulmonary SPIONs delivery accuracy from millimeter to sub-millimeter scale without introducing new materials or additional safety concerns. Integrating this strategy with the macrophage- or cancer-membrane camouflage technologies and with magnetic hyperthermia-immunomodulation offers an immediately testable route to bring SPIONs theranostics from fixed peripheral tumors to the moving lung environment.

### 4.4. Multifunctional Surface Engineering Strategies

**Figure 3:**
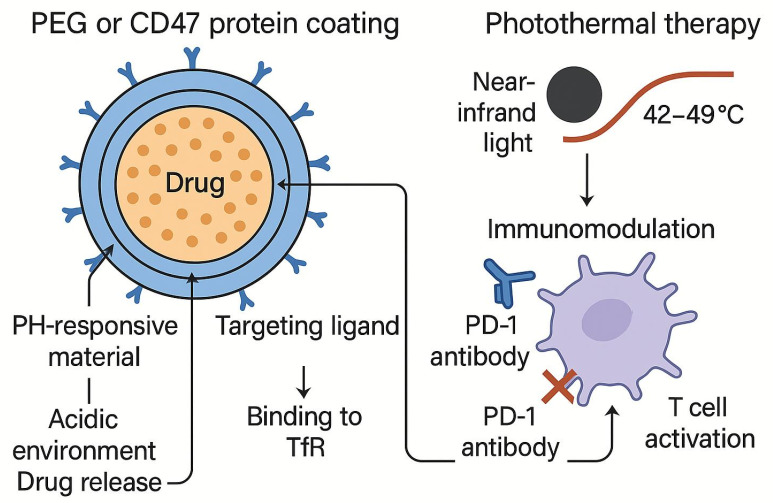
Surface modification strategies and therapeutic applications of SPIONs: (1) Surface functionalization: SPIONs are modified with PEG or CD47 protein coating to enhance immune evasion, while being conjugated with targeting ligands (e.g., transferrin, Tf) and PD-1 antibodies to achieve dual targeting (TfR binding and immune checkpoint blockade). (2) Microenvironment-responsive mechanisms: In the acidic tumor microenvironment (pH 6.5), pH-responsive materials (e.g., PLA-PEG) trigger drug release. Near-infrared irradiation induces photothermal therapy (42–49 °C), synergizing with chemodynamic therapy and immune modulation (e.g., CD8^+^ T-cell activation). (3) Multimodal synergy: The integration of targeted delivery, magnetic hyperthermia, immunotherapy, and microenvironment-responsive drug release enables theranostic applications.

The surface engineering strategy achieves multifunctional integration by precisely regulating the physical and chemical properties of SPIONs (Figure 3), providing an important way to improve their targeting and therapeutic effect in lung cancer treatment. This strategy fully leverages the unique magnetic responsiveness and modifiable surface chemistry of SPIONs, enabling them to better adapt to the tumor microenvironment characteristics and achieve more precise drug delivery as well as multimodal synergistic therapy. Research has demonstrated that SPIONs not only exhibit excellent magnetic responsiveness but also possess abundant surface chemical groups, enabling versatile functionalization. This allows them to integrate multiple therapeutic modalities into a single platform [54,56].

In photodynamic therapy applications, the hypoxic tumor microenvironment often limits reactive oxygen species (ROS) generation efficiency. To address this challenge, Lv et al. [56] developed an innovative nanosystem co-loading the photosensitizer chlorin e6 and ultrasmall SPIONs within a honeycomb-structured MnO_2_ carrier. This system not only enables real-time monitoring of drug distribution through MRI/photoacoustic imaging, but its MnO_2_ component also decomposes in the tumor microenvironment to generate oxygen, thereby partially alleviating local hypoxia and enhancing ROS production efficiency. This “self-oxygen-supplying” design concept offers a novel approach to overcome the limitations of conventional photodynamic therapy. To address the tissue penetration depth limitation in photodynamic therapy, Zhang et al. [67] developed a hollow iron-oxide-hematoporphyrin composite system. This system utilizes ultrasonic energy to trigger the decomposition of endogenous H_2_O_2_, not only partially overcoming the insufficient light penetration issue but also demonstrating therapeutic potential for deep-seated tumors. This combined sonodynamic-magnetothermal therapeutic approach opens new avenues for treating deep-seated lung cancers. In the field of chemodynamic therapy, insufficient tumor microenvironment acidity often compromises treatment efficacy. To address this issue, Shi et al. [54] developed an acid-responsive magnetic nanoplatform (FePt@FeOx@TAM-PEG). This platform achieves drug release through the protonation of tamoxifen (TAM) while simultaneously inhibiting mitochondrial complex I activity and promoting glycolysis, thereby increasing local lactate concentration in tumors. The study also incorporates multimodal imaging technology, providing technical support for real-time treatment monitoring and personalized therapeutic strategies.

Advancing surface engineering strategies are driving progress in the field of “nano-catalytic medicine”. Future research may increasingly focus on developing SPION systems with metabolic microenvironment-adaptive capabilities, achieving more tumor-specific therapies through biomimetic catalytic mechanisms. Meanwhile, exploring the synergistic effects between ferroptosis and immune modulation may offer new opportunities to overcome the limitations of conventional ROS-mediated killing. The development of intelligent platforms integrating diagnosis, treatment, and evaluation functions—enabling real-time imaging monitoring combined with dynamic therapeutic adjustments—will also be a noteworthy direction for future research. These innovations rely not only on breakthroughs in material design but also require a deeper understanding of the molecular mechanisms underlying tumor metabolic reprogramming, thereby advancing treatment modalities from passive drug delivery to active regulation.

## 5. Therapeutic Applications and Mechanisms

### 5.1. Strategies and Mechanisms for Overcoming Physiological Barriers

In the field of targeted therapy for lung cancer, one of the biggest challenges facing drug-delivery systems is how to effectively overcome complex physiological barriers to achieve precise drug delivery. As an important physiological barrier, the tumor microenvironment has a significant impact on the treatment effect due to its characteristic immunosuppressive state. Among them, the dynamic plasticity of tumor-associated macrophages (TAMs) is particularly critical, which accounts for more than 50% of the microenvironment of most solid tumors and mainly exhibits a tumor-promoting M2 phenotype [87,88]. M2 macrophages not only support tumor growth but also help tumors evade immune surveillance by secreting immunosuppressive factors such as IL-10 and TGF-β and promoting angiogenesis [89]. In recent years, it has been found that abnormal activation of the PI3Kγ signaling pathway in macrophages is a key molecular mechanism driving M2 polarization, which provides a new intervention idea for improving the tumor microenvironment by targeting TAMs [90]. However, considering the important role of the PI3K-Akt-mTOR pathway in maintaining normal immune cell function, it is particularly important to develop precise therapeutic strategies that can selectively target PI3Kγ in TAMs without affecting other immune cells.

Superparamagnetic iron-oxide nanoparticles (SPIONs), as a novel delivery vehicle, demonstrate unique advantages in overcoming multiple physiological barriers. Compared with traditional viral vectors and liposomes, SPIONs exhibit superior biocompatibility, lower immunogenicity, and significantly enhanced delivery efficiency due to their excellent dispersibility [91,92]. Particularly noteworthy is the superparamagnetic property of SPIONs, which enables non-invasive targeting under external magnetic field guidance. By precisely controlling magnetic field parameters, drug concentration at tumor sites can be increased several-fold [92]. From the perspective of cellular uptake mechanisms, SPIONs primarily enter cells through endocytosis, a process that not only effectively protects loaded drugs or genes from lysosomal enzyme degradation but also provides sustained therapeutic effects due to their prolonged circulation half-life [92,93]. These combined characteristics make SPIONs an ideal carrier for overcoming tumor physiological barriers.

When SPIONs enter the blood circulation, their interaction with blood components is directly related to the treatment effect and safety. Neutrophils, the most abundant leukocytes in circulation (about 65%), play a central role in host defense, migrate rapidly to sites of inflammation, and participate in the immune response by releasing antimicrobial substances and inflammatory mediators [94]. However, studies have found that insufficiently surface-modified SPIONs tend to aggregate in blood due to magnetic attraction, high surface energy, and van der Waals forces [95]. More notably, when SPIONLA1 and SPIONLA2 are exposed to magnetic fields, they form nanoparticle aggregates both in vitro and in vivo, which can activate neutrophils to release neutrophil extracellular traps (NETs) [69]. NETs crosslink and immobilize SPIONs aggregates through their extracellular chromatin-rich structures, forming stable SPIONs-aggNETs co-aggregates. In extreme cases, this may even lead to microvascular occlusion—a phenomenon requiring particular attention in pulmonary capillary beds [52,96]. These findings highlight the crucial role of SPION surface modification. Experimental evidence demonstrates that proper coating with human serum albumin (SPIONs-LA-HSA) or dextran (SPIONs-DEX) can effectively prevent such undesirable aggregation [76,77,97,98].

Immediately after SPIONs enter the circulation, they are encapsulated by a variety of serum proteins (including complement components, immunoglobulins, fibronectins, and apolipoproteins, etc.) to form a “protein crown” [77]. This natural biomolecular coating enhances the colloidal stability and biocompatibility of nanoparticles [71,72,73], but also accelerates the recognition and clearance of SPIONs by the reticuloendothelial system (RES) [74]. Studies have shown that unmodified SPIONs are mainly taken up by Kupfer cells in the liver and spleen, leading to their rapid clearance in the blood circulation [79]. To prolong cycle time, commonly used strategies include surface modification with polyethylene glycol (PEG) to create an “invisible” coating [99], or direct delivery of SPIONs to tumor-supplied blood vessels by intra-arterial injection, thereby reducing systemic exposure [80,81]. Notably, neutrophils exhibit distinct size-dependent clearance mechanisms for SPIONs: smaller individual SPIONs are primarily eliminated through conventional phagocytosis, while larger aggregates preferentially induce NETosis—a specialized cell death pathway [100]. NETosis activation involves neutrophil elastase (NE) activation, which promotes chromatin decondensation and ultimately leads to NET release [69]. Further research also found that the three-dimensional structural characteristics of SPION aggregates, such as particle size, local concentration, and surface topology, have an important impact on the induction intensity of NETosis [100], and this effect is similar to the inflammatory response mechanism elicited by some biomaterial implants [101,102].

From the perspective of the coagulation system, the interaction of SPION aggregates with NETs may trigger an abnormal coagulation cascade. On the one hand, NE can cleave tissue factor pathway inhibitors (TFPIs), thereby enhancing procoagulant activity [103,104,105]; On the other hand, the histones carried by NETs can directly activate platelets and promote thrombosis [75]. These mechanisms work synergistically to significantly increase the risk of microvascular thrombosis, especially in areas of capillary beds with slow blood flow [52,96]. In addition, the involvement of the complement system further complicates this process: activated complement proteins (such as C3a and C5a) can induce NET formation, which in turn can serve as platforms for complement activation, forming a positive feedback loop [106]. Although some iron-oxide-based contrast agents have been reported to cause hypersensitivity reactions or complement activation [107,108], no significant complement activation-related pseudoallergic (CARPA) reactions have been observed in vitro and in vivo experiments with well-coated SPIONs (e.g., SPIONDEX) [76,78]. In particular, SPIONLA–HSA, which is designed for magnetic drug targeting, has an artificially constructed albumin crown that not only significantly improves colloidal stability but also greatly improves biocompatibility, providing safety for clinical applications.

Based on the existing research evidence, the critical role of SPION surface engineering in overcoming physiological barriers has been well confirmed. In the absence of an external magnetic field, properly coated SPIONs (such as SPIONLA–HSA and SPIONDEX) can maintain a good dispersion state in the plasma or serum environment without inducing adverse reactions such as NETosis [76,77,97,98]. However, once a magnetic field is applied, aggregation occurs in inadequately modified SPIONs (such as SPIONLA1 and SPIONLA2) that significantly alter their surface topological properties, which in turn triggers NET formation and increases the risk of thrombosis [52,69]. These findings provide important guidance for the clinical translation of SPIONs: first, the risk of adverse reactions can be minimized while maintaining magnetic responsiveness by optimizing surface coatings (such as HSA or dextran); Secondly, choosing the appropriate route of administration (such as intra-arterial injection) can improve targeting efficiency. Finally, precise control of magnetic field parameters is a key part of ensuring safe and effective treatment. The systematic integration of these strategies provides a practical solution for SPIONs to break through multiple physiological barriers in targeted therapy for lung cancer, and lays an important foundation for the development of safer and more efficient nanodelivery systems in the future.

### 5.2. Synergistic Immunotherapy

Immune escape is one of the important features of cancer, involving the complex interaction of innate immunity and adaptive immune systems, which together determine tumor progression and treatment response [109]. In recent years, magnetic hyperthermia (MHT) based on superparamagnetic iron-oxide nanoparticles (SPIONs) has shown unique potential in modulating the tumor immune microenvironment. A study by Carter et al. [59] showed that in a mouse model of glioblastoma, intratumoral injection of dextran-coated SPIONs (Perimag-COOH) followed by magnetic hyperthermia significantly increased the proportion of CD8 T-cell infiltration in the tumor while inhibiting tumor growth. The increase in CD8 T-cells is often associated with a favorable prognosis [110], a finding that provides an experimental basis for SPIONs combined with immunotherapy. However, the regulation of the immune microenvironment is complex, and Covarrubias et al. [111] found in a 4T1 breast cancer model that although SPIONs-induced hyperthermia can reduce the infiltration of various immune cells (such as neutrophils, dendritic cells, macrophages, and T-cells), subsequent combined with immune checkpoint inhibitor therapy can reactivate immune cell function, suggesting that the synergistic effect of SPIONs and immunotherapy needs to be precisely designed according to tumor type and microenvironment characteristics. In addition, SPIONs may reverse the immunosuppressive microenvironment by regulating the polarization state of tumor-associated macrophages (TAMs) and converting the tumor-promoting M2 type into the antitumor M1 type [112].

The use of SPIONs in immunotherapy is not limited to hyperthermia effects. Pfister et al. [68] confirmed that human T-cells loaded with SPIONs were not affected in terms of mechanical properties and function, and their antigen-specific activation, proliferation ability, and tumor-killing function were preserved. This property opens up the possibility of SPIONs as T-cell vectors for adoptive cell immunotherapy, such as CAR-T therapy. In terms of induction of immunogenic cell death (ICD), SPIONs can serve as multifunctional platforms loaded with ICD inducers such as oxaliplatin or doxorubicin to enhance drug accumulation at tumor sites through magnetically targeted delivery [113]. Damage-associated molecular patterns (DAMPs) released during ICD can activate inflammatory immune responses, which are more conducive to stimulating systemic antitumor immunity than traditional apoptosis. For example, nanoparticle-encapsulated ICD inducers have shown superior therapeutic effects over free drugs in pancreatic cancer models, and the magnetic targeting properties of SPIONs further enhance this advantage [113].

**Figure 4:**
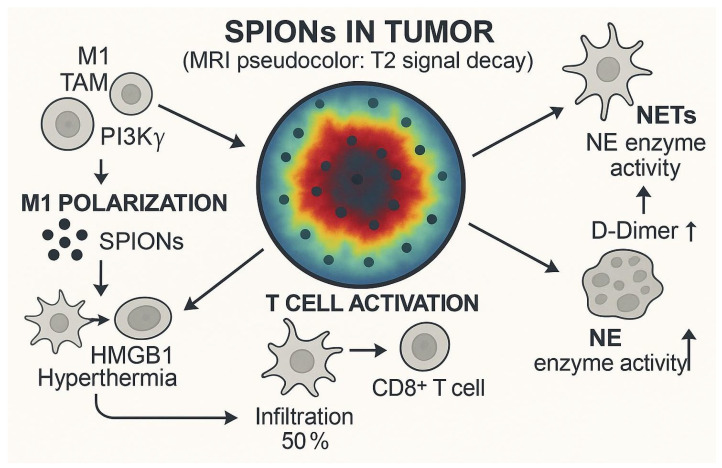
The mechanism by which SPIONs reshape the tumor immune microenvironment: (1) Macrophage polarization regulation: SPIONs promote the polarization of tumor-associated macrophages (TAMs) from tumor-pro-tumor M2 to antitumor M1 by inhibiting the PI3Kγ signaling pathway (red arrow). (2) T-cell activation: Magnetic hyperthermia (MHT) induces tumor cells to release damage-related molecular patterns (HMGB1), promotes CD8 T-cell infiltration (↑ 50%) and activation, and enhances immune response. (3) Neutrophil extracellular trapping nets (NETs) effect: SPIONs aggregates may activate neutrophil elastase (NE), leading to the release of NETs and increased levels of D-dimers, potentially affecting the risk of thrombosis.

Immune checkpoint inhibitors, such as PD-1/CTLA-4 antibodies, have made breakthroughs in a variety of cancers, but still face challenges such as toxicity, low response rates, and high costs [114]. SPIONs offer new ideas to address these issues (Figure 4). For example, the delivery of PD-1 antibodies or siRNAs through nanoparticles can precisely regulate the PD-L1/PD-1 signaling pathway while reducing systemic side effects [115]. In addition, SPIONs targeting circulating T-cells can take advantage of the tumor-homing properties of lymphocytes to enhance drug penetration in deep tumor tissues, thereby reducing therapeutic doses and improving safety [116]. Recent studies have even attempted to bind SPIONs to T-cells in vitro and then guide them to the tumor area through magnetic fields, and preliminary experiments have shown feasibility [117]. These advances suggest that the combination of SPIONs with immunotherapy can not only overcome the limitations of monotherapy but may also achieve more durable antitumor effects through multi-mechanism synergies such as immune microenvironment remodeling, ICD induction, and checkpoint regulation. However, how to optimize the physicochemical properties of SPIONs to balance immune activation and safety, and how to individually design joint protocols are still key directions for future research.

## 6. Translational Medicine Challenges

The translational application of superparamagnetic iron-oxide nanoparticles (SPIONs) in targeted therapy for lung cancer faces multiple critical challenges. While these nanoparticles demonstrate tremendous potential in targeted drug delivery, magnetic hyperthermia, and molecular imaging diagnostics due to their unique physicochemical properties, significant obstacles remain in translating laboratory research into clinical applications.

Regarding manufacturing processes, SPIONs face substantial technical bottlenecks in large-scale production. The 131I-labeled bevacizumab-conjugated paclitaxel-loaded SPIONs (131I-BEV-PTX-SPIONs) developed by Ji et al. [58] showed excellent stability and targeting capability in vitro and in animal experiments, but their complex preparation process limits feasibility for industrial-scale production. Similarly, the targeted drug-delivery system constructed by Ngema et al. [118] through HRH peptide modification achieved a 76.6% tumor suppression rate in lung adenocarcinoma models, but the reproducibility and batch-to-batch consistency of this surface functionalization method still require further optimization. Standardization and scaling-up of these manufacturing processes represent primary barriers to clinical translation.

In terms of targeting efficiency, existing research results show considerable variability. The anti-EGFR-modified SPIONs developed by Shahbazi-Gahrouei et al. [119] demonstrated excellent tumor accumulation characteristics in lung cancer models, but the targeting efficiency observed in preclinical studies is often difficult to replicate in humans. This discrepancy partly stems from the complexity of human physiological environments, where factors such as hemodynamics and respiratory movements are challenging to fully simulate in animal models. Additionally, tumor heterogeneity leading to variations in target expression can significantly impact SPIONs’ targeting efficacy [53].

Safety assessment for clinical applications also presents challenges. Although SPIONs-based sentinel lymph-node biopsy has shown good safety in breast cancer diagnosis (with a detection rate of 99% and only mild adverse reactions) [60], long-term safety data at therapeutic doses remain limited. Particularly in magnetic hyperthermia applications, the localized high temperatures induced by SPIONs may damage normal tissues, and their mechanisms of action on the immune system are not yet fully understood [59,120]. Research by Persano et al. [120] found that the immunological characteristics of tumor cells change after magnetic hyperthermia—while these changes may enhance the killing effect of immune cells, they could also trigger unforeseen immune responses.

In clinical validation, current research data show a clear imbalance between diagnostic and therapeutic applications. The diagnostic field has accumulated substantial clinical evidence, particularly showing significant progress in tumor staging and lymph-node localization. SPIONs-based sentinel lymph-node biopsy technology has demonstrated excellent diagnostic performance in multiple clinical studies, achieving a 99% identification rate in 143 Turkish patients with early-stage breast cancer with only mild adverse reactions [60]. Research from Uppsala University Hospital further confirmed that SPIONs injected 3–15 days preoperatively could consistently detect all sentinel lymph nodes, with axillary signals maintained for up to 28 days [86]. Larger-scale clinical studies (146 patients) showed that the magnetic technique achieved a 99.3% lymph-node detection rate, representing a 0.7% improvement over traditional radiotracers [61]. These results collectively indicate that SPIONs have established reliable clinical application value in tumor diagnostics.

In tumor staging applications, ultrasmall superparamagnetic iron-oxide particles (USPIO) have also shown remarkable performance. In bladder/prostate cancer staging studies, USPIO-MRI combined with diffusion-weighted imaging achieved 93–96% specificity, with three physicians requiring only 9 min to evaluate 75 patients—significantly better than traditional CT examinations [121]. Studies in pediatric cancer patients also showed that intravenous ferumoxytol at a 5 mg Fe/kg dose achieved over 90% accuracy in identifying different types of lymph nodes [82]. These clinical data provide important references for SPIONs applications in lung cancer diagnosis.

However, in stark contrast to diagnostic applications, clinical validation of SPIONs in therapeutic fields lags significantly behind. Currently, only a few clinical trials have explored their therapeutic potential, such as a Phase I study for osteosarcoma (NCT04316091) investigating rotating magnetic field-triggered SPIONs combined with neoadjuvant chemotherapy [122]. While this study provides reference insights for lung cancer treatment, dedicated clinical research for lung cancer remains very limited. Notably, ferumoxytol has demonstrated therapeutic potential in various disease [123] models (including acute myocardial infarction [124,125], cerebral aneurysms [126], and abdominal aortic aneurysms [127]), but these applications have not yet been extended to lung cancer. In neurological diseases, while ferumoxytol-enhanced MRI failed to confirm the association between macrophage-mediated inflammation and migraines [62], its safety in pediatric patients has been preliminarily verified, laying the groundwork for expanding clinical applications.

In summary, the clinical translation of SPIONs in targeted therapy for lung cancer needs to address multiple challenges, such as the preparation process, targeting efficiency, safety evaluation, and clinical validation. Future research should focus on optimizing large-scale production methods, establishing more precise targeted delivery strategies, improving safety evaluation systems, and conducting more clinical trials for lung cancer. Only through these systematic efforts can SPIONs technology truly move from the laboratory to the clinic, bringing new treatment options for lung cancer patients.

## 7. Narrative Comparison: Biomimetic SPIONs vs. Pegylated Liposomes vs. PLGA-PEG Nanoparticles

To objectively position biomimetic SPIONs in the clinical nanomedicine landscape, we benchmarked them against two established nano-platforms: pegylated liposomes (Doxil-like) and PLGA-PEG polymeric nanoparticles (Genexol-PM-like) across six translational axes.

Active targeting. Biomimetic SPIONs inherit native membrane proteins such as integrin αvβ3, CD47, and EGFR, enabling ligand-free homotypic recognition that has been shown to double tumor accumulation compared with non-coated SPIONs in lung cancer models [49,51]. In contrast, liposomes and PLGA particles require post-fabrication conjugation of antibodies or aptamers, introducing additional synthetic steps and batch-to-batch variability [63].

Triggered release. The superparamagnetic core of SPIONs provides an on-demand release mechanism: external alternating magnetic fields raise local temperatures to 42–45 °C, simultaneously activating thermo-sensitive lipids or polymers and accelerating drug diffusion [9,55]. Neither Doxil nor Genexol-PM offers an external trigger; release relies solely on passive diffusion or slow polymer erosion, respectively [51].

Real-time imaging. SPIONs possess intrinsic T_2_ MRI contrast (r_2_ ≈ 150–200 mM^−1^ s^−1^) and can be quantified with emerging magnetic particle imaging at ~1 mm resolution, allowing same-particle theranostics without additional labels [14,15]. Liposomal or PLGA systems must co-encapsulate separate imaging agents, typically reducing drug-loading capacity by >10% [63].

Dose-limiting toxicities. The newly identified liability for biomimetic SPIONs is NETosis-related micro-thrombosis: inadequately coated particles aggregate under magnetic fields, provoking neutrophil extracellular traps and elevated D-dimer levels in pulmonary capillaries [52,96]. Pegylated liposomes are limited by hand–foot syndrome and acute infusion reactions, while PLGA particles carry a known risk of complement activation-related pseudo-allergy (CARPA) [107,108].

Regulatory precedent. Only ferumoxytol, a diagnostic iron-oxide nanoparticle, has reached Phase III approval; no therapeutic SPION has yet cleared efficacy gates [60]. Doxil (1995) and Genexol-PM (2001) enjoy fully validated CMC and safety packages, giving them a considerable head-start in regulatory familiarity [63].

Scalability and batch consistency. Macrophage or neutrophil membranes rely on primary animal cells, introducing 10^2^–10^3^% inter-donor variability in membrane-protein density [41,43]. Cancer-cell membranes can be harvested from GMP master cell banks, but universal membrane-quality specifications (orientation, purity, DNA carry-over) remain undefined [51,53]. Conversely, lipids for Doxil and PLGA monomers for Genexol-PM are pharma-grade excipients with established supply chains and <5% batch-to-batch variation [63].

In summary, biomimetic SPIONs trade manufacturing and safety complexity for unique magnetic triggerability and built-in imaging, a profile unmatched by existing liposomal or polymeric systems. Future trials should exploit this theranostic bonus to implement adaptive, image-guided dose-escalation strategies, thereby differentiating SPIONs from the purely drug-delivery value proposition of Doxil or Genexol-PM.

## 8. Conclusions and Perspectives

Biomimetic SPIONs have emerged as a theranostic powerhouse for lung cancer, integrating cell-membrane stealth, magnetic precision, and real-time imaging into one nanoplatform. Despite manufacturing and regulatory hurdles, the field is poised for quantum-leap studies that move beyond incremental animal models. We propose the following concrete, priority-ranked directions.

Inhalable Magnetic Aerosols. Leverage approved ferumoxytol as the iron core and nebulize CCM-SPIONs (100–150 nm) via a vibrating-mesh inhaler; preliminary lung deposition in mice reached 65% of the total dose [45,60]. Add mannitol osmotic adjuster to prevent aggregation during aerosolization—an approach already validated for liposomal inhalers [63]. Checkpoint-Free Immuno-Chemodynamic Combo. Replace antibody-based PD-1 blockade with CDT-inducible ICD: load SPIONs with oxaliplatin + dihydroartemisinin; magnetic heating (42 °C, 20 min) synchronizes ROS burst + HMGB1 release, converting “cold” tumors to “hot” without systemic checkpoint toxicity [9,68,109]. TAM-Specific PI3Kγ CRISPR Cargo. Use macrophage-membrane SPIONs to deliver PI3Kγ sgRNA plasmid (pH-sensitive histidine-modified coating); magnetic guidance concentrates particles in M2-dominant niches, achieving gene knockdown 70% vs. 25% with free plasmid [90]. Human Lung-on-Chip NET-Thrombosis Threshold. Establish a NET-formation safety index: expose primary neutrophils in a microfluidic alveolar-capillary chip to incremental SPION doses (0.1–1 mg Fe mL^−1^) ± 0.5 T field; define “no-observed-adverse-effect level” (NOAEL) as NET area < 10% lumen and D-dimer < 0.5 μg mL^−1^ [52,96]. GMP-Ready Cell-Bank Consortium. Create a public repository of cryopreserved master cell banks (A549-CCM, THP-1-MM) with standardized membrane integrity index (MII)—vesicle size 100–120 nm, zeta potential −20 ± 5 mV, CD47 outside-out >90%—to eliminate batch-to-batch CV > 15% observed in primary-cell preparations [51,53].

By tackling these specific, measurable milestones, the next wave of biomimetic SPION research can transition from high-impact papers to high-impact prescriptions, ultimately delivering precision pulmonary theranostics to the clinic.

## Figures and Tables

**Table 1:** Functional comparison of different membrane types.

Membrane Type	Major Membrane-Protein Components	Targeting Characteristics	Immune-Evasion Capability	Circulation Time	Preparation Difficulty	Application Scenarios	References
Macrophage membrane	PSGL-1, LFA-1, VLA-4	VCAM-1-mediated inflammation/tumor-targeting	Moderate (retains partial self-recognition signals)	Moderate (days)	Moderate	Inflammatory sites, tumor microenvironment targeting	[40,41]
Neutrophil membrane	CD11b/CD18, CD62L	Integrin-ICAM-1-mediated blood-air barrier penetration	Strong (natural immune-evasion properties)	Short (hours)	Difficult	Lung targeting, acute inflammation therapy	[42,43,44,45]
Cancer-cell membrane	Tumor-specific antigens (e.g., EGFR), integrin αvβ3	Homologous targeting (same tumor type)	Strong (expresses “don’t-eat-me” signals)	Moderate (days)	Moderate	Primary tumor and metastasis therapy	[49,51]

Note: Scoring criteria (0–4 scale). (1). Immune-evasion capacity: 0 = rapid MPS clearance; 4 = CD47-mediated “don’t-eat-me” or natural immune silence. Macrophage: score 2 (partial CD47 retention) [20,41]. Neutrophil: score 3 (low NETosis when single particles) [45,52]. Cancer cell: score 3 (high CD47 copy number ≈ 1200 μm^−2^) [20,50]. (2). Preparation difficulty: 0 = single-step synthesis; 4 = multi-step primary-cell isolation with <50% yield. Macrophage: score 2 (bone-marrow harvest + culture) [41]. Neutrophil: score 3 (short lifespan, activation-sensitive) [43]. Cancer cell: score 2 (cell-line expansion, but DNA-removal step required) [51,53]. (3). Circulation half-life: derived from plasma Fe concentration (ICP-MS) post-i.v. injection in mice [41,45,51].

**Table 2:** Summary of studies on biomimetic SPIONs for lung cancer therapy.

Cell-Membrane Type	Functional Modification	Therapeutic Mechanism	Model	Key Findings	Conclusion	References
Neutrophil membrane	poly(sialic acid)-octadecylamine	Neutrophil-mediated delivery	Mouse lung cancer model	Enhanced drug delivery to lung tumor site	Neutrophil membrane improves lung targeting	[45]
Cancer-cell membrane	Curcumin + DOX co-loading	Homologous targeting + MDR reversal	Esophageal cancer model (extensible to lung)	Effective inhibition of drug-resistant tumor growth	CCM enhances tumor-specific accumulation	[51]
No membrane (MnO_2_ shell)	Ce6 photosensitizer + O_2_ generation	Self-oxygenated PDT + MRI/PA imaging	Mouse lung cancer model	Alleviated hypoxia, enhanced ROS production	Overcomes hypoxia-induced PDT resistance	[56]
Neutrophil exosome hybrid	Transferrin (Tf) conjugation	Magnetic targeting + exosome homing	Mouse lung-metastasis model	Enhanced lung accumulation via Tf and neutrophil tropism	Dual targeting improves lung retention	[57]
Lung cancer cell membrane	Tumor-associated antigens preserved	Homologous targeting + immune evasion	Mouse lung cancer model	Enhanced tumor enrichment, evaded immune clearance	Cancer membrane improves tumor-specific targeting	[65]
Mesoporous silica shell	Fe_3_O_4_@mSiO_2_ core–shell	High paclitaxel loading via mesopores	In vitro/vivo tumor models	Improved hydrophobic drug-loading and release	Mesoporous shell enhances drug compatibility	[66]
Hollow SPIONs	Hematoporphyrin + US-triggered H_2_O_2_ decomposition	SDT + MHT synergy	Mouse tumor model	Overcame light penetration limit, enhanced deep tumor therapy	Combined SDT-MHT effective for deep tumors	[67]
T-cell membrane	SPIONs loaded into human T-cells	T-cell function preservation + magnetic navigation	Human T-cells in vitro	T-cell activation and cytotoxicity unaffected	SPIONs can serve as T-cell carriers for immunotherapy	[68]

## Data Availability

Not applicable.

## Abbreviations

CDT	hemodynamic therapy
SPIONs	Superparamagnetic iron-oxide nanoparticles
MHT	magnetic hyperthermia therapy
MRI	magnetic resonance imaging
MPI	magnetic particle imaging
EPR	enhanced permeability and retention
MDR	multidrug resistance
NETs	neutrophil extracellular traps
GMP	Good Manufacturing Practice
PLA-PEG	poly(lactic acid)-poly(ethylene glycol)
Tf	transferrin
ICD	immunogenic cell death
TAMs	tumor-associated macrophages
iPSC	induced pluripotent stem cell
